# Protective effect of calcitonin on lumbar fusion-induced adjacent-segment disc degeneration in ovariectomized rat

**DOI:** 10.1186/s12891-015-0788-7

**Published:** 2015-11-09

**Authors:** Chang-Cheng Liu, Fa-Ming Tian, Zhuang Zhou, Peng Wang, Yu Gou, Heng Zhang, Wen-Ya Wang, Yong Shen, Ying-Ze Zhang, Liu Zhang

**Affiliations:** Orthopaedic Research Institution of Hebei, Third Hospital of Hebei Medical University, Shijiazhuang, 050017 P.R. China; Medical Research Center, North China University of Science and Technology, Tangshan, 063000 P. R. China; Department of Orthopedic Surgery, Affiliated Hospital of North China University of Science and Technology, No. 73 Jianshe South Rd., Tangshan, Hebei 063000 P.R. China; Department of Pathology, School of Basic Medical Sciences, North China University of Science and Technology, Tangshan, 063000 P. R. China

**Keywords:** Lumbar fusion, Adjacent-segment disc degeneration, Osteoporosis, Calcitonin, Micro-computed tomography

## Abstract

**Background:**

Intervertebral disc (IVD) degeneration and pathological changes in the spinal cord are major causes of back pain. In addition to its well-established anti-resorptive effect on bone, calcitonin (CT) potentially exerts protective effects on IVD degeneration in ovariectomized rats. However, possible therapeutic effects of CT on lumbar fusion-induced adjacent-segment disc degeneration (ASDD) have not been investigated yet. In this study, we examined the effects of CT on IVD degeneration adjacent to a lumbar fusion in ovariectomized rats.

**Methods:**

Posterolateral lumbar fusion (PLF) at L4–5 was performed 4 weeks after ovariectomy (OVX) or sham surgery in female Sprague–Dawley rats. Following PLF + OVX, rats received either salmon CT (OVX + PLF + sCT, 16 IU/Kg/2d) or vehicle (OVX + PLF + V) treatment for 12 weeks; the remaining rats were divided into Sham + V, OVX + V, and PLF + V groups. Fusion status was analyzed by manual palpation and radiography. Adjacent segment disc was assessed by histological, histomorphometric, immunohistochemical analysis. L6 vertebrae microstructures were evaluated by micro-computed tomography.

**Results:**

Histological analysis showed more severe ASDD occurred in OVX + PLF + V rats compared with the OVX + V or PLF + V groups. CT treatment suppressed the score for ASDD, increased disc height, and decreased the area of endplate calcification. Immunohistochemical staining demonstrated that CT decreased the expression of collagen type-I, matrix metalloproteinase-13, and a disintegrin and metalloproteinase with thrombospondin motifs-4, whereas it increased the expression of collagen type-II and aggrecan in the disc. Micro-computed tomography indicated that CT increased bone mass and improved the microstructure of the L6 vertebrae.

**Conclusions:**

These results suggest that CT can prevent ASDD, induce beneficial changes in IVD metabolism, and inhibit deterioration of the trabecular microarchitecture of vertebrae in osteoporotic rats with lumbar fusion.

## Background

Lumbar fusion is increasingly performed as the treatment of choice for various degenerative disorders of the lumbar spine [1–3]. Adjacent-segment disc degeneration (ASDD) is a problematic complication that can occur following lumbar spinal fusion [4, 5]. Within 5 years of lumbar fusion surgery, the clinical incidence of symptomatic ASDD was reported as 5.2–18.5 % [6]. Thus, the prevention of ASDD after spinal fusion is a challenge that needs to be addressed.

Large numbers of patients undergoing lumbar fusion are the late-middle-aged and elderly population being treated for osteoporosis, which is characterized by a systemic impairment of bone mass and microarchitecture that increases the propensity of fragility fractures due to rapid bone absorption. It would be interesting if a treatment for osteoporosis could also be effective for preventing ASDD after spinal fusion. As an anti-resorptive agent, calcitonin (CT) acts on osteoclasts to inhibit bone resorption and is currently widely used in clinical practice to treat osteoporosis and other diseases involving accelerated bone turnover [7, 8]. In addition to its anti-resorptive effect on bone, a previous study revealed that CT reduced biochemical markers of cartilage degradation [9]. Moreover, our previous study confirmed the protective effects of CT on intervertebral disc (IVD) degeneration in osteoporotic rats [10]. However, to our knowledge, there are no available data on the effect of CT on ASDD induced by lumbar fusion in the ovariectomized rat.

The structure of the endplate facilitates important biomechanical and nutritional functions. Biomechanically, the endplate is subjected to significant loads during activities of daily living as the trunk muscles contract to stabilize posture. Nutritionally, the endplate is the primary pathway for transport between vertebral capillaries and cells within the disk nucleus [11]. Endplate sclerosis, which impacts the biomechanical properties, has long been thought to play a role in disc degeneration by decreasing nutrient availability to the disc; disc degeneration occurs concomitantly with marked architectural bony changes on the endplate surface [12]. Moreover, as the gateway of nutrient supply, the vertebral endplate is essential to maintain the integrity and function of the avascular IVD, with increased degeneration in the adjacent IVD being associated with greater endplate thickness [13].

Extracellular matrix (ECM) metabolism plays a key role in the integrity and function of theintervertebral disc. Collagen type II (Coll-II) and aggrecan content is crucial to proper disc function. During degeneration, the synthesis of ECM components changes, leading to an increase in the synthesis of Collagen type I (Col-I) and decreased production of aggrecan. Linked tothis is an increased expression of matrix-degrading molecules, including matrix metalloproteinases (MMPs), aggrecanases, and a disintegrin and metalloproteinase with thrombospondin motifs (ADAMTS) −1, −4, −5, −9, and −15, all of which are produced by native disc cells [14]. For further insight into the mechanism of action of CT on disc ECM metabolism, we explored whether aggrecan, Col-II, Col-I, MMP-13, and ADAMTS-4 participate in this process.

The aim with the study was to determine whether CT, a commonly prescribed anti-osteoporotic agent, could not only prevent bone loss, but also retard IVD degeneration adjacent to the fused lumbar spine in the ovariectomized rat, by detecting the fusion status, bone mass and microstructure of the vertebrae, and by evaluating the intervertebral disc degeneration through histology, disc height and endplate calcification measurement, and immunohistochemistry (IHC) analysis for aggrecan, Col-II, Col-I, MMP-13, and ADAMTS-4.

## Methods

### Animal handling and study design

A total of 50, 3-month-old, female Sprague–Dawley rats (260 ± 16 g, Peking University Animal Center, Beijing, China) were used in this study. All animals were housed at 20 °C with a 12-h light/dark cycle and free access to water and food. All experimental procedures were approved by the Institutional Animal Care and Use Committee of North China University of Science and Technology.

All rats received either Sham (*n* = 20) surgery or bilateral ovariectomy (OVX) (*n* = 30). Four weeks after surgery, all but ten10 rats from each group underwent Posterolateral lumbar fusion (PLF) at L4–L5 [15]. Rats received both OVX and PLF were treated by either (1) saline vehicle treatment (OVX + PLF + V, *n* = 10); or (2) subcutaneous injection of salmon CT (Novartis AG, Switzerland) at a dose of 16 IU/kg per 2 days (OVX + PLF + sCT, *n* = 10). The remaining rats from each group received saline vehicle treatment and were divided into three groups of 10 each: Sham + V, OVX + V, and PLF + V. The weight of each animal was recorded weekly and the quantity of drug was adjusted according to the new weight recordings. After 12 weeks of treatment, all animals were sacrificed.

After scarification, radiographic evaluation and manual palpation were performed to evaluate the lumbar fusion, and then the segment of the rat L3–L6 spine was removed and scanned by dual energy X-ray absorptiometry to determine bone mineral density (BMD). Five samples from the L6 segment from each group were prepared for micro-computed tomography analysis, while the remaining samples were used for paraffin sectioning, followed by van Gieson staining and immunohistochemical staining.

### Evaluation of lumbar fusion

First, an anteroposterior plain soft radiograph (DR7500 System, Kodak, USA) of the spine was performed to evaluate spinal fusion, and then imaging scores were graded using the criterion described by O’Loughlin et al. [16], as follows: 0, no bone; 1, poor new bone formation; 2, moderate new bone formation and definite pseudarthrosis; 3, moderate new bone formation and possible pseudarthrosis; 4, good new bone formation and probable fusion; 5, definite fusion. After radiographic assessment, fusion status in the lumbar spine was manually palpated, as described by Abe et al. [17]. Each spine was assessed and graded as fixed or not fixed in a blinded manner. The lack of motion was defined as successful fusion.

### Bone mineral density assessment

BMD of L3–L6 was performed on the anteroposterior plane by dual energy X-ray absorptiometry using a densitometer (QDR Discovery, Hologic, Bedford, MA, USA) operating at high-resolution mode, using specialized software for small animals supplied by the equipment manufacturer.

### Micro-computed tomography analysis

Three-dimensional (3D) analysis was performed on the trabeculae of the cancellous tissue of the L6 vertebrae. Samples were scanned by SkyScan 1076 Micro-CT (SkyScan, Aartselaar, Belgium) with a resolution of 36 μm per voxel. The data were collected at 100 kV and 100 μA. The volume of interest was defined as being restricted to an inner cylinder with a 1.5-mm diameter and a 3-mm length (1–4 mm proximal to the growth plate), excluding the cortex, which is consistent with our previous study. The 3D images and bone histomorphometry indices were calculated by the built-in software within the machine, followed by comparing the difference between two groups. The following 3D morphometric parameters were calculated to describe the bone mass and microstructure: BMD, the percent bone volume (BV/TV), trabecular thickness (Tb.Th), trabecular number (Tb.N), trabecular separation (Tb.Sp), and structural model index (SMI).

### Histological and histomorphometric analysis of the L5-6 segments

The L5–L6 segments of the lumbar spine were fixed in 10 % neutral buffered formalin, decalcified in 10 % EDTA-2Na for 3 months, split down at the mid-sagittal plane with a scalpel, and embedded in paraffin. Samples were then cut into 5-μm-thick parasagittal plane sections, placed on microscope slides and stained with van Gieson (VG) stain for light microscopic examination. Images were captured on a BX53 microscope system (Olympus, Japan). The degenerative changes in L5-6 segment were assessed using the disc degeneration assessment scoring system (Table 1) described by Wang et al. [18].

**Table 1:** Lumbar intervertebral disc degeneration assessment scoring system

Score	Nucleus pulposus	Annulus fibrosus	Osteophyte
0	Bulging gel with abundant notochordal cells	Compactfibrous lamellas	Absence
1	Notochordal cells loss; chondrocyte-like cells emergence	Proliferation offibrocartilaginous tissue and loss of nuclear–annular border	Appearance
2	Focal mucoid degeneration; clefts	Fissures in annulusfibrosis	Overgrowth
3	Diffuse mucoid degeneration and clefts throughout nucleus		

Disc height measurements were taken from the caudal aspect of the growth plate of L5 to the cranial aspect of the growth plate of L6 on histological samples from the L5-6 segments. For each image, an average of three measurements were made from three areas of the disc space for one section from each rat: one from the anterior, one from the central, and one from the posterior side [19]. The thickness of endplate was measured from the cranial growth plate to the border between the nucleus pulposus and the endplate in the VG staining. The ratio of calcified area to the total endplate area were determined by an average of 10 measurements made in the endplate, with the semi-automatic digitizing system mentioned above. All measurements were performed using a digital image analysis system (BX53, Olympus, Japan).

### Immunohistochemical analysis

Immunohistochemistry for Coll-I, Coll-II, aggrecan, MMP-13 and ADAMTS-4 was performed using SA1024 SABC-POD kit (Boster Co., Ltd., Wuhan, China) and Kit-0017 DAB detection kit (Maxim Co., Ltd. Fuzhou, China). Tissue sections from L5 to L6 segments were deparaffinized in xylene and rehydrated in a reverse-graded series of ethanol. After antigen retrieval, quenching of endogenous peroxidase and blocking of non-specific binding, sections were incubated with primary antibodies (Boster, Wuhan, China) overnight at 4 °C. Then, sections were incubated in biotinylated secondary antibodies at 37 °C for 20 min, followed by incubation for 20 min with SABC-Peroxidase solution at 37 °C, and developing using a DAB kit under the microscrope. Sections were counter stained with hematoxylin. Samples appearing yellow or brownish-yellow were considered as positive staining. All sections were semi-quantitatively analyzed by Image Pro Plus (IPP) version 6.0 software, and the integrated optical density (IOD) was measured from staining of six fields for each section, using one section per rat, on the images at 400× magnification. The average IOD from three observers was the final observation result and used for statistical analysis.

### Statistical analysis

All data were analyzed using SPSS 19.0 software and results are expressed as mean ± standard deviation. The statistical significance between groups was estimated using one-way analysis of variance and the Fisher’s protected least significant difference test. The results of the histological and radiography scores were analyzed using the Kruskal–Wallis test. *P* values less than 0.05 were considered statistically significant.

## Results

### Lumbar fusion

**Fig. 1:**
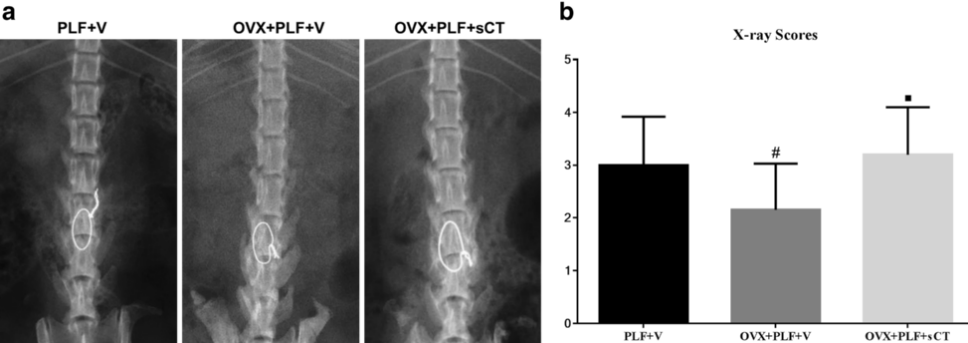
Representative radiographic images (**a**) and X-ray scores (**b**) of the three groups underwent lumbar fusions at 12 weeks post-PLF. The PLF was performed at the L4-5 segment using an intertransverse process fusion with an autologous iliac bone graft and spinous-process wire fixation. The OVX + PLF + sCT group showed a higher radiographic density than the OVX + PLF + V group. Note: ^#^ *P* = 0.043 vs. PLF + V group; ^■^ *P* = 0.013 vs. OVX + PLF + V group

As shown in Fig. 1, a lower radiographic density was observed in OVX + PLF + V rats compared with the PLF + V and OVX + PLF + sCT groups. The radiographs revealed that bone formation activity had increased in the OVX + PLF + sCT group compared with the OVX + PLF + V group, with a significantly greater amount of fusion mass. The radiographic scores indicated a delayed fusion process in OVX + PLF + V (2.15 ± 0.88) rats, which was significantly lower than the PLF + V (3.00 ± 0.92) (*P* = 0.043) and OVX + PLF + sCT (3.20 ± 0.9) groups (*P* = 0.013).

### BMD of L3–L6 vertebrae

The same trends in the differences in BMD of the L3–L6 vertebrae were seen between any two of all five groups (Table 2). Rats in the OVX + V (*vs.* Sham + V: *P* < 0.001, *P* = 0.001, *P* < 0.001, *P* = 0.003) and OVX + PLF + V groups (*vs.* PLF + V: *P* < 0.001, *P* = 0.002, *P* = 0.024, *P* = 0.015) exhibited significantly lower BMD compared with the Sham + V and PLF + V groups; while OVX + PLF + sCT rats showed significantly higher BMD compared with OVX + PLF + V rats (*P* < 0.001, *P* = 0.001, *P* = 0.004, *P* = 0.011).

**Table 2:** BMD values among the five groups (Mean ± SD, g/cm^2^, *n* = 10)

Group	Sham + V	OVX + V	PLF + V	OVX + PLF	OVX + PLF + CT
L3	0.3399 ± 0.0090	0.2995 ± 0.0250*	0.3201 ± 0.0110	0.2907 ± 0.0288*^#^	0.3219 ± 0.0271^■^
L4	0.3533 ± 0.0180	0.3089 ± 0.0214*	0.3418 ± 0.034	0.3039 ± 0.0221*^#^	0.3377 ± 0.036^■^
L5	0.3768 ± 0.0131	0.3295 ± 0.0223*	0.3561 ± 0.0351	0.3272 ± 0.0186*^#^	0.3580 ± 0.0309^■^
L6	0.3983 ± 0.0198	0.3681 ± 0.0191*	0.3808 ± 0.0248	0.3507 ± 0.0198*^#^	0.3718 ± 0.0199^■^

Note: *P < 0.05 *vs.*Sham + V group; ^#^ *P* < 0.05 *vs.* PLF + V group; ^■^ *P* < 0.05 *vs.*OVX + PLF + V group;

### Microstructural parameters of the L6 vertebrae

**Fig. 2:**
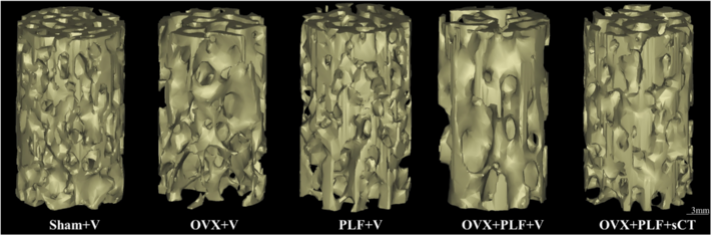
Representative micro-CT images of the L6 vertebrae in different groups. The morphology of the trabeculae was rod-shaped, and the width of the canal between trabeculae was increased in the OVX + V and OVX + PLF + V groups compared with the Sham + V group. The OVX + PLF + sCT group showed increased trabecular thickness and decreased canal width between the trabeculae compared with the OVX + PLF + V group

Three-dimensionally reconstructed micro-CT images are shown in Fig. 2. Micro-computed tomography showed that BV/TV, Tb.Th, and Tb.N were significantly lower, and Tb.Sp and SMI were significantly higher in the OVX + V (*vs.* Sham + V: *P* < 0.001*, P* < 0.001, *P* = 0.002, *P* < 0.001, *P* < 0.001) and OVX + PLF + V groups (*vs.* Sham + V: *P* < 0.001(all parameters); *vs.* PLF + V: *P* < 0.001, *P* < 0.001, *P* = 0.001, *P* < 0.001, *P* < 0.001) compared with the Sham + V and PLF + V groups. CT-treated rats had markedly higher BV/TV, TB.Th, and Tb.N and lower Tb.Sp and SMI compared with the OVX + PLF + V group (*P* < 0.001, *P* = 0.039, *P* = 0.048, *P* = 0.043, *P* < 0.001) (Table 3).

**Table 3:** Microarchitecture parameters of vertearal body by micro-CT analysis (Mean ± SD, *n* = 5)

Group	Sham + V	OVX + V	PLF + V	OVX + PLF + V	OVX + PLF + sCT
BMD (mg/cc)	496.04 ± 24.01	397.06 ± 21.81*	480.21 ± 18.94	370.83 ± 17.58*^#^	420.47 ± 21.48^■^
BV/TV (%)	42.62 ± 3.30	28.33 ± 2.03*	40.68 ± 0.94	26.26 ± 3.29*^#^	31.72 ± 4.13^■^
Tb.Th (μm)	83.68 ± 3.31	59.55 ± 5.28*	79.11 ± 2.05	57.14 ± 5.67*^#^	64.17 ± 3.46^■^
Tb.N (mm^−1^)	5.27 ± 0.3	4.45 ± 0.4*	4.96 ± 0.5	4.25 ± 0.4*^#^	4.65 ± 0.5^■^
Tb.Sp (μm)	110.83 ± 13.47	157.49 ± 18.80*	124.84 ± 14.84	168.48 ± 17.48*^#^	142.41 ± 18.35^■^
SMI	0.57 ± 0.27	1.42 ± 0.14*	0.62 ± 0.12	1.54 ± 0.13*^#^	1.20 ± 0.18^■^

Note: *P<0.05 *vs.*Sham + V group; ^#^ *P*<0.05 *vs.* PLF + V group; ^■^ *P*<0.05 *vs.*OVX + PLF + V group;

### Histological and histomorphometric findings in L5-6 vertebral discs

**Fig. 3:**
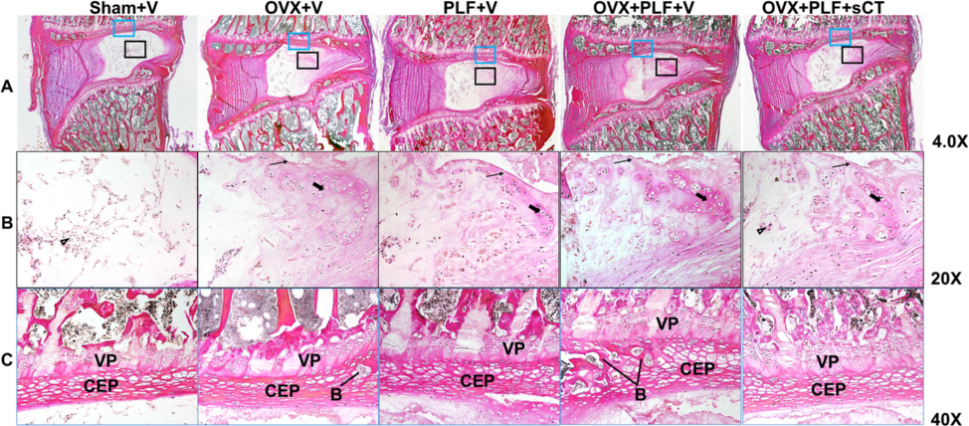
Van Gieson staining of the L5-6 segments of the lumbar spine in different groups. **a** Low magnification. **b** The degenerative changes in the nucleus pulposus. The blank arrow indicates notochord cells, the thin arrow indicates mucoid degeneration of the nucleus pulposus. and the thick arrow indicates doublets of chondrocyte-like cells that appeared in the nucleus pulposus. **c** The degenerative changes in the middle cartilage endplate. Bony tissues were more prevalent in the OVX + PLF + V group compared with the Sham + V, OVX + V, and PLF + V groups. VP, vertebral physis; CEP, cartilage endplate; B, bony tissues

The Sham + V group appeared to exhibit abundant notochordal cells and surrounding extracellular matrix in the nucleus pulposus, well-arranged outer annulus fibrosus, and the endplate was rich in hyaline cartilage. The OVX + V, PLF + V, and OVX + PLF + V groups experienced degeneration of the nucleus pulposus, annulus fibrosus, and endplate. Loss of cellularity of notochordal cells and their replacement by chondrocyte-like cells were found in the nucleus pulposus, and the matrix displayed mucoid degeneration. Loss of collagen, proliferation of fibrocartilaginous tissue, and disruption of the nuclear-annular border were observed in the annulus fibrosus. The endplate exhibited large areas of calcification to different degrees. These mineralized tissues became more obvious in the deep zone of the middle cartilage endplate. All degenerative changes were more obvious in the OVX + PLF + V group compared with the OVX + V or PLF + V groups (Fig. 3).

**Fig. 4:**
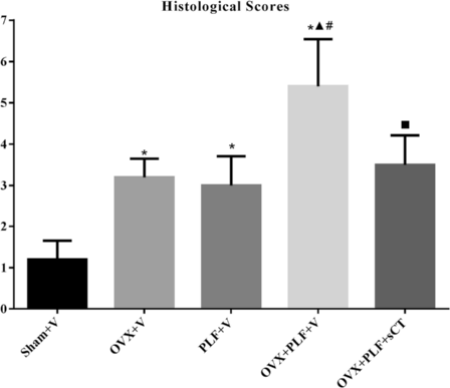
Histological scores of the L5-6 intervertebral disc at 12 weeks after PLF. the histological score in the OVX + V, PLF + V, OVX + PLF + V, and OVX + PLF + sCT groups were markedly higher than the Sham + V group; the highest value was found in the OVX + PLF + V group, which was significantly different with OVX + PLF + sCT group. Note: *P < 0.001 vs. Sham + V group; ^▲^ *P* < 0.001 vs. OVX + V group; ^#^ *P* < 0.001 vs. PLF + V group; ^■^ *P* = 0.001 vs. OVX + PLF + V group

Accordingly, the histological score in the OVX + V, PLF + V, OVX + PLF + V, and OVX + PLF + sCT groups were markedly higher (*P* < 0.001, *P* < 0.001, *P* < 0.001, *P* < 0.001) than the Sham + V group; the highest value was found in the OVX + PLF + V group, which was significantly different compared with any other group (*vs.* all group: *P* < 0.001) (Fig. 4).

**Fig. 5:**
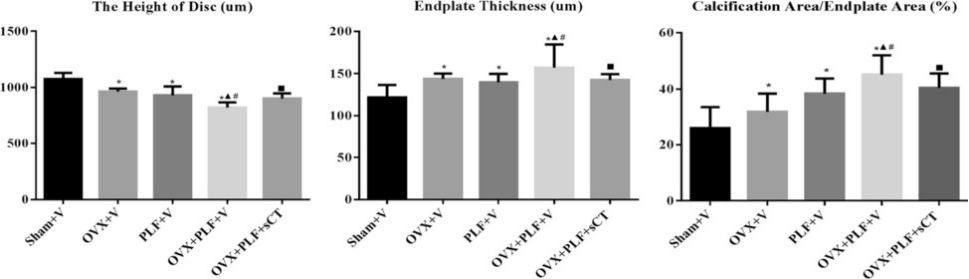
The disc height , endplate thickness and the ratio of calcified area to endplate area of the endplate of L5-6 disc. Compared with the Sham + V group, the disc height in the OVX + V, PLF + V, and OVX + PLF + V groups were all decreased and the opposite trends were found in endplate thickness and ratio of calcified area to total endplate area. The OVX + PLF + V group showed the most serious degenerative changes, with the lowest disc height, highest endplate thickness, and largest ratio of calcified area to the total endplate area. sCT treated rats showed significantly higher disc height, lower endplate thickness, and decreased ratio of calcified area to the total endplate area, compared with the OVX + PLF + V group. Note: *P < 0.05 vs. Sham + V group; ^▲^ *P* < 0.05 vs. OVX + V group; ^#^ *P* < 0.05 vs. PLF + V group; ^■^ *P* < 0.05 vs. OVX + PLF + V group

The disc height in the OVX + V, PLF + V, and OVX + PLF + V groups were all decreased (*P* < 0.0019*, P* < 0.001, *P* < 0.001) compared with the Sham + V group, and the opposite trends were found in endplate thickness (*P* < 0.001, *P* = 0.005, *P* < 0.001) and ratio of calcified area to total endplate area (*P* = 0.048, *P* < 0.001, *P* < 0.001). The OVX + PLF + V group showed the most serious degenerative changes, with the lowest disc height, highest endplate thickness, and largest ratio of calcified area to the total endplate area (*vs.* Sham + V: *P* < 0.001, *P* < 0.001, *P* < 0.001). sCT treatment markedly prevented these degenerative changes, indicated by the significantly higher disc height, lower endplate thickness, and decreased ratio of calcified area to the total endplate area, compared with the OVX + PLF + V group (*P* = 0.003, *P* = 0.014, *P* = 0.001) (Fig. 5).

### Immunohistochemical staining

**Fig. 6:**
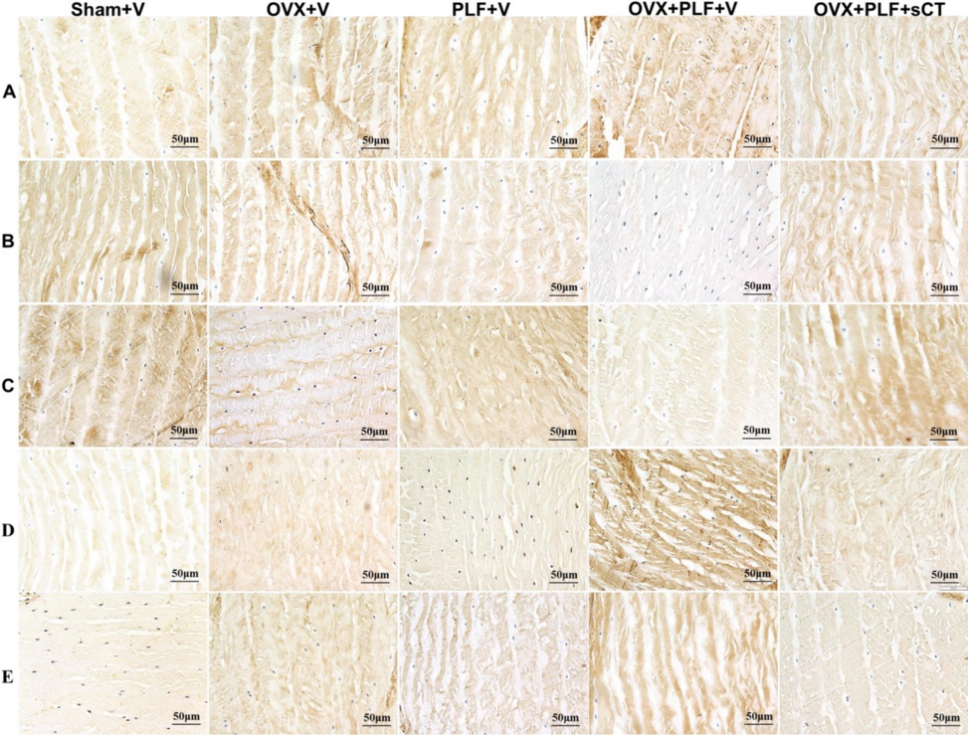
Immunohistochemistry assay for **a** Col-I, **b** Col-II, **c** Aggrecan, **d** MMP-13, and **e** ADAMTS-4 in the nucleus pulposus in different groups (40×). Immunohistological analysis showed that the protein expression of Col-II and Aggrecan in the OVX + PLF + sCT group was higher than that in the OVX + PLF + V group. Col-I-, MMP-13-, and ADAMTS-4-positive staining in the nucleus pulpous was weaker in the OVX + PLF + sCT group compared with the OVX + PLF + V group

**Fig. 7:**
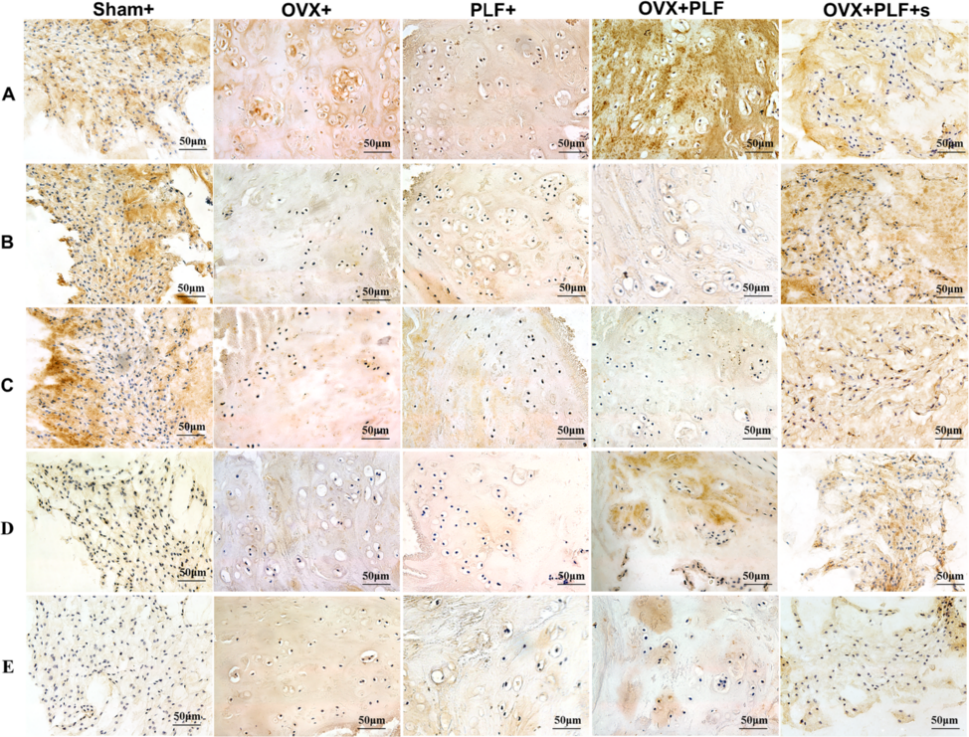
Immunohistochemistry assay for **a** Col-I, **b** Col-II, **c** Aggrecan, **d** MMP-13, and **e** ADAMTS-4 in the annulus fibrosus in different groups (40×). Col-I-, MMP-13-, and ADAMTS-4-positive staining in the annulus fibrosus was weaker in the OVX + PLF + sCT group compared with the OVX + PLF + V group. Col-II- and Aggrecan-positive staining in the annulus fibrosus was stronger in the OVX + PLF + sCT group compared with the OVX + PLF + V group

As shown in Figs. 6 and 7, in the Sham + V group, aggrecan and Col-II expressions were extensive in the nucleus pulposus and annulus fibrosus, with weak expression of MMP-13, ADAMTS-4, and Col-I compared with the OVX + V, PLF + V, and OVX + PLF + V groups. OVX + PLF + sCT rats exhibited stronger aggrecan and Col-II expressions and weak MMP-13, ADAMTS-4, and Col-I expressions compared with OVX + PLF + V rats.

**Fig. 8:**
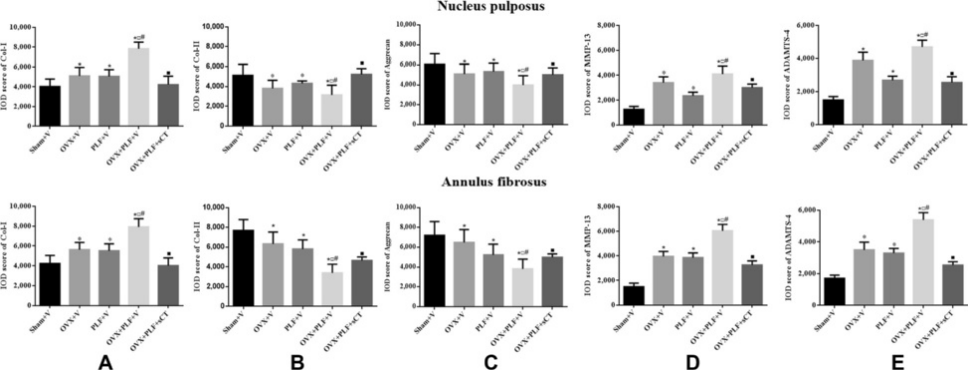
The IOD of Col-I (**a**), Col-II (**b**), Aggrecan (**c**), MMP-13 (**d**), and ADAMTS-4 (**e**) in the nucleus pulposus and annulus fibrosus. Compared with the Sham + V group, the levels of aggrecan and Col-II were significantly lower, and MMP-13, ADAMTS-4, and Col-I were significantly higher in the OVX + V, PLF + V, and OVX + PLF + V groups in the nucleus pulposus and annulus fibrous. OVX + PLF + sCT group showed significantly higher levels of Col-II and aggrecan and lower levels of MMP-13, ADAMTS-4, and Col-I in comparison with OVX + PLF + V group. Note: *P < 0.05 vs. Sham + V group; ^□^ *P* < 0.05 vs. OVX + V group; ^#^ *P* < 0.05 vs. PLF + V group; ^■^ *P* < 0.05 vs. OVX + PLF + V group

Accordingly, the quantitative IOD values confirmed these differences. Compared with the Sham + V group, the levels of aggrecan and Col-II were significantly lower, and MMP-13, ADAMTS-4, and Col-I were significantly higher in the OVX + V, PLF + V, and OVX + PLF + V groups in the nucleus pulposus and annulus fibrous (Fig. 5). CT can significantly increase the levels of Col-II and aggrecan (NP: *P* < 0.001, *P* = 0.001; AP: *P* = 0.001, *P* = 0.004), and decrease the levels of MMP-13, ADAMTS-4, and Col-I (NP: *P* < 0.001, *P* < 0.001, *P* < 0.001; AP: *P* < 0.001, *P* < 0.001, *P* < 0.001) (Fig. 8).

## Discussion

To our knowledge, this is the first study to determine the effects of CT on ASDD induced by posterolateral lumbar fusion in an ovariectomized rat model. Based on the histological and histomorphometric results, the present study demonstrates that osteoporosis induced by ovariectomy exacerbates posterolateral lumbar fusion-induced ASDD. More importantly, in addition to preserving vertebral BMD and trabecular bone microstructure, treatment with CT can prevent histological degeneration in the adjacent disc, as well as the thickening of the endplate and the increased ratio of calcified endplate area to total endplate area. CT can also inhibit the changes in ECM metabolism by counteracting the upregulation of MMP-13, ADAMTS-4, and Col-I and inhibition of aggrecan and Col-II expression in the IVDs of OVX + PLF + V rats.

ASDD after spinal fusion has been well documented in previous studies. Phillips et al. [20] reported that spinal fusion, created using methylmethacrylate and wire, caused IVD degeneration at adjacent segments 6 months after surgery in rabbits. Higashino et al. [21] showed ASDD occurred 12 months after spinal fusion surgery. These degenerative changes in the adjacent discs after spinal fusion were confirmed in the present study. In contrast, in our previous study we confirmed IVD degeneration occurred in OVX rats. The data provided in the present study demonstrates that rats receiving both OVX and PLF surgeries displayed more serious ASDD than those receiving only OVX or PLF, indicating that the combination of OVX and PLF exacerbates ASDD. Further study is needed to determine whether there is a synergistic effect between or only a cumulative effect of the two causative factors.

The present study showed an increased ratio of calcified endplate area to total endplate area and greater endplate thickness in OVX, PLF, and OVX + PLF + V groups, with the OVX + PLF + V group showing much higher values than the other two groups. These histomorphometric changes were partially prevented by CT treatment, thus indicating a protective effect of CT against endplate degeneration, making it beneficial for maintaining the biomechanical environment and nutrition status of the IVD.

For further insight into the mechanism of action of CT on disc ECM metabolism, we explored whether aggrecan, Col-II, Col-I, MMP-13, and ADAMTS-4 participate in this process. Compared with Sham + V rats, decreased expression levels of Col-II and aggrecan, along with increased levels of Col-I, MMP-13, and ADAMTS-4, were observed in OVX + PLF + V rats. In contrast, CT treatment thoroughly reversed these changes in expression levels, indicating that the protective effects of CT on ASDD in this model were, at least in part, due to modulation of the synthesis and degradation of ECM.

In addition to the direct evidence supporting the preventive effect of CT on the histological changes of ASDD, we also found decreased BMD and deterioration of the vertebral trabecular bone microstructure were observed in OVX + PLF + V rats, which were successfully counteracted by CT treatment. However, the relationship between BMD and disc degeneration is still debatable. Some previous studies have supported that higher BMD of the vertebral body is associated with more severe adjacent disc degeneration [22], whereas lower lumbar spine BMD was associated with less severe disc degeneration [23]. It has also been noted that relative estrogen deficiency likely contributes to accelerated disc degeneration in post-menopausal women [24, 25]. The reason for this contradiction may be the biological function of estrogen. Estrogen deficiency, as was similarly induced in the OVX model in the present study, is not only the main cause of bone loss, but is also accompanied by a progressive proinflammatory status, evidenced by increased systemic interleukin (IL)-1, IL-6, and tumor necrosis factor-α [26], which are potentially important in the pathogenesis of intervertebral disc degeneration [27, 28]. Furthermore, the structural integrity of the vertebral body might play an important role in the progression of disc degeneration, whereby aberrant loading can give rise to localized tissue injury [29]. The altered trabecular structure of the osteoporotic spine leads to an increased vulnerability of its biomechanical characteristics and reduction of load resistance. Therefore, it should be reasonable to consider that the preservation of the microstructure of the vertebral body trabecular bone may contribute to the preventive effects of CT on ASDD in OVX + PLF + V rats.

It is interesting that, although it causes ASDD, lumbar fusion was enhanced by CT treatment in the present study. This result is similar to other previous studies [30, 31]. Babat et al. [30] found that rates of L5–L6 posterolateral fusion with autografts in rabbits were 68 % when CT was administered compared with 56 % in controls. Liu et al. [31] reported that CT enhanced lumbar spinal fusion in a New Zealand rabbit model. Based on the histological and histomorphometric results shown in the present study, the enhanced lumbar fusion does not affect the protective effect of CT on the IVD. It should be highlighted that CT may be used as a dual-effect treatment for not only lumbar fusion, but also ASDD in osteoporosis.

## Conclusions

Taken together, our results indicate that CT treatment inhibits ASDD in OVX rats, with the underlying mechanism possibly being associated with regulation of the expression of aggrecan, Col-II, Col-I, MMP-13, and ADAMTS-4, and, conversely, preservation of BMD and trabecular bone microstructure of the adjacent vertebrae. Remarkably, this beneficial effect on the IVD is accompanied with an enhancement of lumbar fusion. Though further study was needed to confirm whether the dosage of CT used in the present study, which was effective in reducing bone turnover and partially preventing cancellous bone loss in the estrogen-depleted skeleton [32, 33], is safe for clinical patients, CT may be a potentially effective treatment for ASDD in postmenopausal osteoporotic patients.

## Abbreviations

ASDD: adjacent-segment disc degeneration
IVD: intervertebral disc
PLF: posterolateral lumbar fusion
OVX: ovariectomy
BMD: bone mineral density
VG: van Gieson
Coll-I: collagen type I
Coll-II: collagen type II
MMP-13: matrix metalloproteinase-13
ADAMTS-4: a disintegrin and metalloproteinase with thrombospondin motifs-4
IOD: integrated optical density
BV/TV: percent bone volume
Tb.Th: trabecular thickness
Tb.N: trabecular number
Tb.Sp: trabecular separation
SMI: structural model index
